# Mesoscopic modeling the interaction of two attached-wall cavitation bubbles^☆^

**DOI:** 10.1016/j.ultsonch.2025.107358

**Published:** 2025-04-16

**Authors:** Weidong Gan, Shicheng Li, Xiaolong He, Dianguang Ma

**Affiliations:** a School of Navigation, Wuhan University of Technology, Wuhan 430063, China; b Department of Civil and Architectural Engineering, KTH Royal Institute of Technology, Stockholm 10044, Sweden; c State Key Laboratory of Hydraulics and Mountain River Engineering, Sichuan University, Chengdu 610065, China; d Tianfu Yongxing Laboratory, Chengdu 610000, China; e Hubei Key Laboratory of Inland Shipping Technology, Wuhan 430063, China; f Tianjin Research Institute for Water Transport Engineering, Key Laboratory of Engineering Sediment, Ministry of Transport, Tianjin 300456, China

**Keywords:** Attached-wall cavitation, Interaction dynamics, Heat flux characteristic, Lattice Boltzmann method

## Abstract

A hybrid thermal lattice Boltzmann cavitation model based on a nonorthogonal framework is developed to investigate the interaction of two attached-wall cavitation bubbles. The interaction modes are systematically analyzed, with an emphasis on how varying contact angles influence the flow and temperature distributions, as well as the evolution of wall heat flux under strong and weak interaction conditions. Bubbles formed on the hydrophobic surface display increased contact radius and greater curvature radii compared to those on the hydrophilic wall, leading to greater volumes but weaker collapse intensity. The growth rate of the bubble equivalent radius for the weak interaction modes consistently follows the relation $U\propto \sqrt{2p_{\infty }/3\rho_{l}}$. Additionally, bubble coalescence occurs at the interface regions along the hydrophobic surface, altering the final collapse dynamics and resulting in distinct temperature and velocity distributions. Finally, the instantaneous heat flux characteristics are explored. Due to differences in the contact points motion rate and microjet angle with the solid wall, the peak value and number of heat flux peaks vary on walls with different wettability.

## 1 Introduction

Liquid hydrogen (LH_2_) has garnered significant attention due to its environmentally friendly by-products and high energy density relative to weight [1]. Thus, LH_2_ is a potential propellant for commercial space launch systems. Nevertheless, when LH_2_ passes via the injection nozzle or turbo-pump system with high speed, the liquid pressure decreases, potentially causing cavitation, along with oscillations and erosion [[1], [2], [3], [4]]. Since the operating temperature of LH_2_ is near its critical temperature, the thermal effects are crucial and can significantly influence the cavitation dynamics [5]. For these reasons, the underlying mechanics of LH_2_ cavitation bubbles interaction and evolution have long intrigued researchers, making it a prominent area of study.

Previous studies determined that the surface wettability and the microstructure significantly influence the attached-wall bubble evolution [[6], [7], [8], [9]]. Naudé & Ellis first studied the cavitation evolution of attached-wall bubbles based on experiments and the potential flow model [6]. Recently, Hupfeld et al. explored the expansion and implosion of bubbles adhered to the wall with high capillary numbers [10]. They paid attention to the influence of liquid viscosity on the contact line and microlayer evolution. However, the bubble collapse in the final period is hard to observe via high-speed photograph techniques owing to the bubble cloud’s formation.

Cavitation bubbles inherently exist in nature and industrial applications with a bubble cluster mode, and the interactions between bubbles and between bubbles and walls are complex [[11], [12], [13], [14], [15], [16], [17], [18]]. Research on LH_2_ primarily concentrated on cavitation flow [[1], [2], [3],^5^], with little attention given to the development of the thermal field at the bubble scale. This oversight may affect bubble implosion modes and associated jetting phenomena, and leads to different bubble clusters. The movement of cavitation bubbles and the pressure gradient between the vapor phase and the surrounding liquid cause significant fluctuations in the adjacent flow field, thereby affecting the evolutionary dynamics of nearby bubbles. The two cavitation bubbles’ interaction represents a fundamental and critical issue in exploring the dynamics of multiple cavitation bubbles. Numerous studies have investigated two bubbles interacting in an unbound region [[18], [19], [20], [21], [22], [23], [24], [25], [26]]. During the in-phase growth of two isolated bubbles, they migrate and collapse toward each other. During the out-of-phase interaction, it become more intricate, often involving bubble rupture and reversal of jets. If the starting distance between the two nuclei is adequately small, the surfaces of the approaching bubbles (inner walls) flatten, and the film between them may rupture. Eventually, the cavitation bubbles coalesce. However, most previous studies have concentrated on the cavitation bubble interaction in the free region, ignoring the interaction between two bubbles adhered to the wall.

Earlier studies investigated the attached-wall cavitation evolution via numerical simulations. Lauer et al. numerically studied attached-wall cavitation bubbles [8]. They indicated that the bubble collapse characteristics depend on the non-dimensional bubble-wall distance, $\gamma =d/r_{\max}$, where d denotes the distance from the center of the bubble to the wall, and $r_{\max}$ denotes the maximum bubble radius. For γ<0, a pin-like shape is reported in shrinkage period, which Koukouvinis et al. linked it to the accumulation of momentum at the contact point [27]. Recently, Saini et al. demonstrated that the pin-like collapse results in jets moving away from the wall [7], thus leading to the formation of a vortex ring, and intensifies convection in the vicinity of the wall. Nevertheless, the abovementioned studies have focused on the single attached-wall cavitation bubble evolution. The interaction behaviors of two cavitation bubbles areinfluenced by both the wall properties and the adjacent cavitation bubbles have not been investigated yet. Especially, the wettability of the wall impacts the bubble spreading behavior, thereby influencing the dynamic evolution of cavitation bubbles.

So far, no single method has been universally effective for cavitation problems across all scales, leading to the use of different models [8,28,29]. Based on their underlying assumptions, these models are classified into the homogeneous equilibrium model and the nonequilibrium mass transfer model [29]. However, both approaches necessitate interface tracking or capturing techniques, such as the volume-of-fluid or level-set methods. Additionally, the complex nonlinear and time-dependent behavior arising from phase change, as well as heat and mass transfer, demands substantial computational resources. The pseudopotential model has become one of the most widely used lattice Boltzmann method (LBM) multiphase flow models due to its efficient handling of complex boundary conditions and low computational cost [30,31]. In present model, the pseudopotential force is introduced to simulate the interaction between phases, resulting in spontaneous phase separation, removing the need for interface-tracking models. This model has been widely used for simulating complex phenomena, such as bubble dissolution [32], cavitation [[33], [34], [35], [36], [37], [38], [39]], and drying in porous media [40,41]. However, most cavitation studies based on the isothermal LBM or the passive scalar thermal LBM model have not considered the evolution of the surface tension with temperature. The variation of the flow and temperature fields with temperature have been studied based on the double-distribution functions (DDF) and the hybrid thermal pseudopotential models [30,42]. Since pressure is obtained using the equation of state (EOS), the local density variation reflects the local pressure variation. The wettability has a significant effect on the collapse intensity of cavitation bubbles moving in the vicinity of the wall, and cavitation bubbles near a hydrophobic wall collapse faster and with greater intensity [43,44]. Our recent study investigated the whole evolution progression of cavitation bubbles affixed to the wall [45]. The study results indicate that the collapse becomes significantly more vigorous as the surface transitions from a hydrophobic to a hydrophilic state. This is due to the fact that the bubble on the hydrophobic wall exhibits greater curvature and is penetrated by a re-entrant jet of greater volume compared to that on the hydrophilic wall.

Our recent study [25] established an LBM cavitation model that can simulate the entire process of evolution of the cavitation bubble adherent to the wall. This paper introduces a novel hybrid thermal lattice Boltzmann model for cavitation, which is then employed to explore the dynamic interactions between two wall-adherent cavitation bubbles. The velocity and multiphase distributions are calculated using a nonorthogonal multiple-relaxation-time (MRT) pseudopotential LBM, while the temperature distribution is derived from the macroscopic temperature equation solved via the fourth-order Runge–Kutta algorithm. The unit conversion method in the LBM and the physical units based on the Carnahan–Starling (C–S) EOS parameters are introduced [46]. The effect of wettability on mode transition range and collapse intensity is examined. Furthermore, its influence on transient heat flux is investigated, along with proposing the underlying mechanism. This study advances our understanding of enhancing heat exchange efficiency via cavitation in LH_2_ flow.

In the following description, the hybrid thermal LB cavitation model in section 2 is introduced, and is verified by a bubble collapse in the unbound region. Section 3 investigates the interaction dynamics and heat flux characteristics of two cavitation bubbles attached to walls. Finally, Section 4 outlines the conclusions.

## 2 Model description

### 2.1 Nonorthogonal pseudopotential LB model

The non-orthogonal MRT collision operator is employed to enhance numerical robustness during the final shrinkage phase, characterized by pronounced interface distortion, while also reducing spurious currents along the interfaces. Since we only replace the orthogonal matrix with a nonorthogonal matrix in the hydrodynamic model to improve computational robustness along with the other elements of the hydrodynamic framework are consistent with our previous studies [45,47]. In this part, only a brief description of the governing equation is provided. The detailed descriptions of the hydrodynamic model can be found in our previous studies [45,47]. The governing particle distribution function used for hydrodynamic simulation is defined as [48]:

(1) $$f_{i}^{*}\left(x,t\right)=f_{i}\left(x,t\right)-\sum_{j}{\overset{¯}{\Lambda }}_{i,j}\left(f_{j}-f_{j}^{\mathrm{eq}}\right){|}_{\left(x,t\right)}+\frac{\Delta t}{2}\left(\overset{¯}{F_{i}}\left(x,t\right)+\overset{¯}{F_{i}}\left(x+e_{i}\Delta t,t+\Delta t\right)\right)$$

where $f_{i}^{*}\left(x,t\right)$ and $f_{i}\left(x,t\right)$ represent the post-collision and pre-collision particle density distribution functions in the *i*-th velocity direction, with **x** and t indicating the spatial and temporal coordinates. Δt is the time increment. $e_{i}$ denotes the discrete velocity vector, $f_{i}^{\mathrm{eq}}$ is the equilibrium distribution function, and $\overset{¯}{F_{i}}$ corresponds to the external force term.

In Eq. (1), the MRT collision operator can be expressed as $\Lambda =M^{-1}SM$, where S is a diagonal matrix of relaxation parameters, M is the nonorthogonal transformation matrix, defined as [49]:

(2) $$M=\left[\begin{array}{ccc} \begin{array}{c} \begin{array}{c} 1\\ 0\\ 0 \end{array}\\ \begin{array}{c} 0\\ 0\\ 0 \end{array}\\ \begin{array}{c} 0\\ 0\\ 0 \end{array} \end{array} & \begin{array}{ccc} \begin{array}{c} \begin{array}{c} 1\\ 1\\ 0 \end{array}\\ \begin{array}{c} 1\\ 1\\ 0 \end{array}\\ \begin{array}{c} 0\\ 0\\ 0 \end{array} \end{array} & \begin{array}{c} \begin{array}{c} 1\\ 0\\ 1 \end{array}\\ \begin{array}{c} 1\\ -1\\ 0 \end{array}\\ \begin{array}{c} 0\\ 0\\ 0 \end{array} \end{array} & \begin{array}{c} \begin{array}{c} 1\\ -1\\ 0 \end{array}\\ \begin{array}{c} -1\\ 1\\ 0 \end{array}\\ \begin{array}{c} 0\\ 0\\ 0 \end{array} \end{array} \end{array} & \begin{array}{ccc} \begin{array}{c} \begin{array}{c} 1\\ 0\\ -1 \end{array}\\ \begin{array}{c} 1\\ -1\\ 0 \end{array}\\ \begin{array}{c} 0\\ 0\\ 0 \end{array} \end{array} & \begin{array}{c} \begin{array}{c} 1\\ 1\\ 1 \end{array}\\ \begin{array}{c} 2\\ 0\\ 1 \end{array}\\ \begin{array}{c} 1\\ 1\\ 1 \end{array} \end{array} & \begin{array}{ccc} \begin{array}{c} \begin{array}{c} 1\\ -1\\ 1 \end{array}\\ \begin{array}{c} 2\\ 0\\ -1 \end{array}\\ \begin{array}{c} 1\\ -1\\ 1 \end{array} \end{array} & \begin{array}{c} \begin{array}{c} 1\\ -1\\ -1 \end{array}\\ \begin{array}{c} 2\\ 0\\ 1 \end{array}\\ \begin{array}{c} -1\\ -1\\ 1 \end{array} \end{array} & \begin{array}{c} \begin{array}{c} 1\\ 1\\ -1 \end{array}\\ \begin{array}{c} 2\\ 0\\ -1 \end{array}\\ \begin{array}{c} -1\\ 1\\ 1 \end{array} \end{array} \end{array} \end{array} \end{array}\right]$$

Compared to the orthogonal transformation matrix, the non-orthogonal transformation **M** matrix has more zero elements. According to the study by Fei et al. [49], it requires 15 % less computational effort compared to the orthogonal moment LBM and exhibits better numerical stability.

ρ and u represent the macroscopic density and velocity, which can be computed as:

(3) $$\rho =\sum_{i}f_{i},\rho u=\sum_{i}f_{i}e_{i}+\frac{1}{2}F\Delta t$$

### 2.2 Thermodynamics simulation

In the absence of viscous dissipation, the energy equation simplifies to: [42,50]:

(4) $$\rho c_{v}\frac{Ds}{Dt}=\nabla ∙\left(\lambda \nabla T\right)$$

where s denotes the entropy, λ represents the thermal conductivity. By applying the Maxwell relations and the chain rule, the temperature field can be derived from the macroscopic temperature function as:

(5) $$Tds=c_{v}dT+T{\left(\frac{\partial p_{\mathrm{EOS}}}{\partial T}\right)}_{\rho }d\left(\frac{1}{\rho }\right)$$

where $c_{v}$ represents the specific heat at constant volume. Consequently, the thermal diffusivity is computed as $\alpha =\lambda /\rho c_{v}$. The governing temperature function is then expressed as [51]:

(6) $$\rho c_{v}\frac{DT}{Dt}=\nabla ∙\left(\lambda \nabla T\right)-T{\left(\frac{\partial p_{\mathrm{EOS}}}{\partial T}\right)}_{\rho }\nabla ∙u$$

Eq. (6) can be solved using the fourth-order Runge–Kutta method, with

(7) $$T\left(x,t+\Delta t\right)=T\left(x,t\right)+\frac{\Delta t}{6}\left(h_{1}+2h_{2}+2h_{3}+h_{4}\right)$$

where $h_{1}$, $h_{2}$, $h_{3}$, and $h_{4}$ are calculated as

$$h_{1}=M\left(T\left(x,t\right)\right),h_{2}=M\left(T\left(x,t\right)+\frac{\Delta t}{2}h_{1}\right)$$

(8) $$h_{3}=M\left(T\left(x,t\right)+\frac{\Delta t}{2}h_{2}\right),h_{4}=M\left(T\left(x,t\right)+{\Delta th}_{3}\right)$$

M(T) is the partial derivative of temperature T with time t, and is expressed as [[52], [53], [54]]:

(9) $$M\left(T\right)=\partial_{t}T=-u∙\nabla T+\frac{1}{\rho c_{v}}\nabla ∙\left(\lambda \nabla T\right)-\frac{T}{\rho c_{v}}{\left(\frac{\partial p_{\mathrm{EOS}}}{\partial t}\right)}_{\rho }\nabla ∙u$$

The second-order central difference scheme is utilized to approximate the first and second derivatives in Eq. (9) with

(10) $$\frac{\partial \varphi }{\partial x_{i}}=\sum_{j=1}^{8}\frac{\omega_{j}e_{j}∙i\left[\varphi \left(x+e_{j}\Delta t\right)-\varphi \left(x-e_{j}\Delta t\right)\right]}{6c_{s}^{2}\Delta t}$$

(11) $$\frac{\partial^{2}\varphi }{\partial x_{i}\partial x_{i}}=\sum_{j=1}^{8}\frac{\omega_{j}\left[\varphi \left(x+e_{j}\Delta t\right)-\varphi \left(x-e_{j}\Delta t\right)\right]}{3c_{s}^{2}{\Delta t}^{2}}$$

where φ represents a scalar field, and i denotes the unit vector in the *i-*th direction.

### 2.3 Model validation

#### 2.3.1 D2 law

**FIG. 1:**
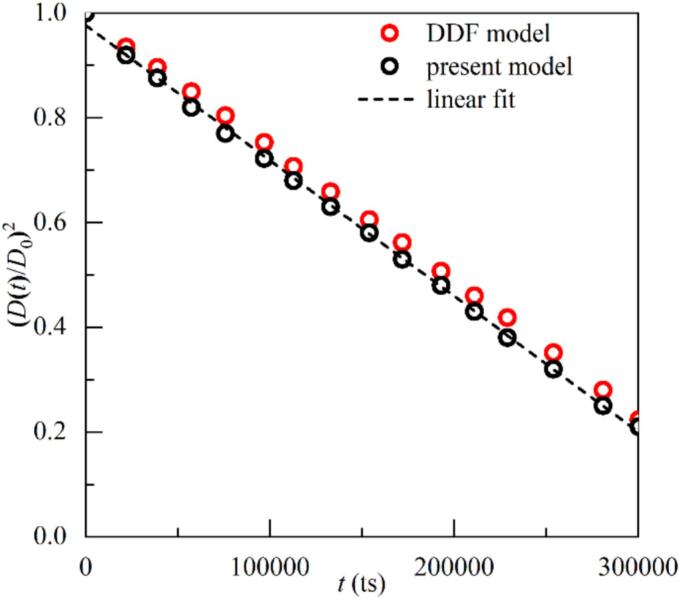
The relationship between the square of the dimensionless diameter ${\left(D\left(t\right)/D_{0}\right)}^{2}$ and time t.

The *D2* law describes a linear reduction in the squared normalized diameter of a droplet as it evaporates over time, is used to verify the accuracy of the thermodynamic simulation in the present model. A droplet with an initial diamater of $D_{0}=60lu$ is placed at the center of a 200lu×200lu square computational domain. Periodic boundary conditions are applied to the flow fields on all four sides, while the constant temperature boundary is utilized for the temperature field simulation with $T_{\infty }=T_{c}$. The initial droplet temperature is set as $T_{l}=0.86T_{c}$, while the surrounding vapor temperature is set as $T_{g}=T_{c}$ to initiate evaporation. Additionally, the simulation results obtained from the DDF model we proposed earlier are also presented here for comparison [21]. Fig. 1 shows the correlation between ${\left(D\left(t\right)/D_{0}\right)}^{2}$ and t, where D(t) represents the droplet diameter at a given time t. The linear fit indicates that the droplet diameter squared diminishes proportionally with time *t*, which is consistent with the *D2* law and supports the simulation results from the DDF model.

#### 2.3.2 Bubble evolves in the unbound region and near-wall region

**Fig. 2:**
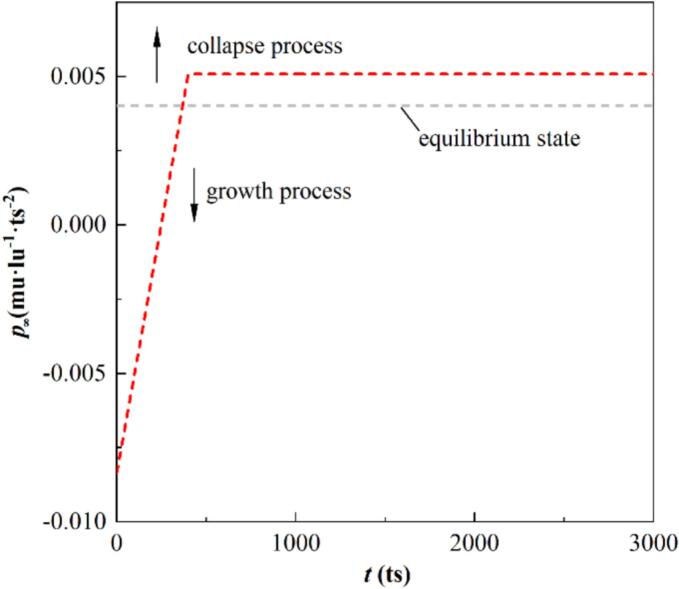
Variation of the pressure boundary.

To verify the proposed model, the complete evolution of a single cavitation bubble in an unconfined domain is numerically simulated. The computational domain is defined as $l_{x}=l_{y}=400lu$, where lu represents the length unit. A vapor nucleus with an initial radius of $r_{0}=2$ lu is placed at the center of the computational domain. Non-equilibrium pressure and constant temperature conditions are applied to all boundaries. The pressure boundary is set as illustrated in Fig. 2, derived from the linearization of pressure for t≥0μs in Bremond’s study [55]. The bubble nucleus expands as long as the liquid pressure remains lower than the vapor phase pressure. When the liquid pressure rises to match the boundary pressure, the bubble undergoes collapse. The initial temperature is set as $T_{\mathrm{ini}}=T_{\infty }=0.5T_{c}$, with the initial density ratio $\rho_{l}/\rho_{v}\approx 720$. The vapor–liquid viscosity ratio is $\nu_{v}/\nu_{l}=15$, with $\tau_{v}=1.0625$ and $\tau_{l}=0.5375$.

The Rayleigh-Plesset (R-P) equation is used here for quantitative validation, which is described as follows [56]:

(12) $$\ln\left(\frac{r_{\infty }}{r}\right)\left({\dot{r}}^{2}+r\ddot{r}\right)-\frac{1-\left(\frac{r_{\infty }}{r}\right)}{2}{\dot{r}}^{2}=\frac{1}{\rho_{l}}\left(p_{v}-p_{\infty }-\frac{2\nu }{r}\dot{r}-\frac{\sigma }{r}\right)-\Omega \dot{r}\sqrt{t}$$

Here, *r* represents the bubble radius, and $r_{\infty }=207.8lu$ is the distance from the bubble center to the pressure boundary. For optimal fitting, this value is selected based on $l_{x}/2$ and the radius of a circle whose area is equivalent to that of the computational domain. In addition, $\dot{r}$ and $\ddot{r}$ denote the first and second derivatives of r. $p_{v}$ represents the average vapor phase pressure, calculated at each timestep from the LBM simulation. The thermal effect term Ω is [56]

(13) $$\Omega =\frac{{\left(\rho_{v}L\right)}^{2}}{\rho_{l}^{2}c_{p}T_{\infty }\alpha^{0.5}}$$

where L represents the vaporization latent heat, calculated as

(14) $$L=h_{v}-h_{l}.$$

where $h_{l}$ and $h_{g}$ are the saturated liquid and the vapor enthalpy. In the case of the C–S EOS, the enthalpy h is given by

(15) $$h=c_{v}T-\rho +\frac{p}{\rho }.$$

**Fig. 3:**
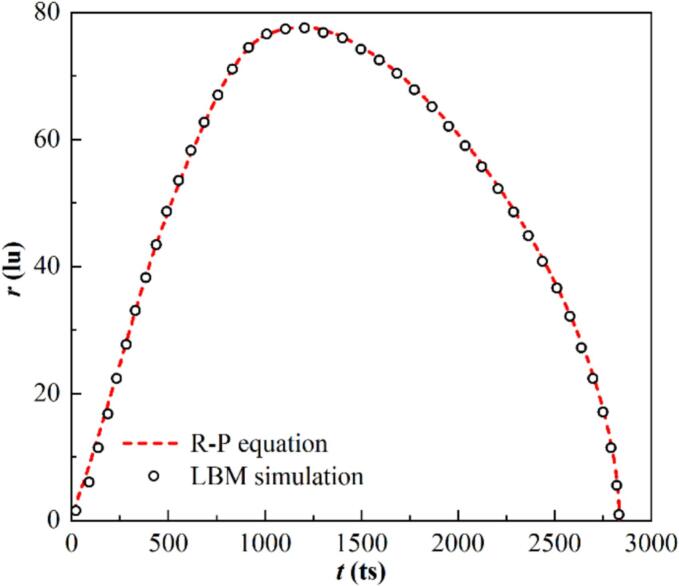
A comparison of the bubble radius evolution process as obtained from both the R–P model and LBM simulation.

Eq. (12) is solved using the fourth-order Runge–Kutta method. The evolution of the bubble radius, as shown in Fig. 3, is obtained from the LBM simulation and compared with theoretical predictions. The simulation results exhibit only minor discrepancies, with a maximum difference of approximately 3 %.

**Fig. 4:**
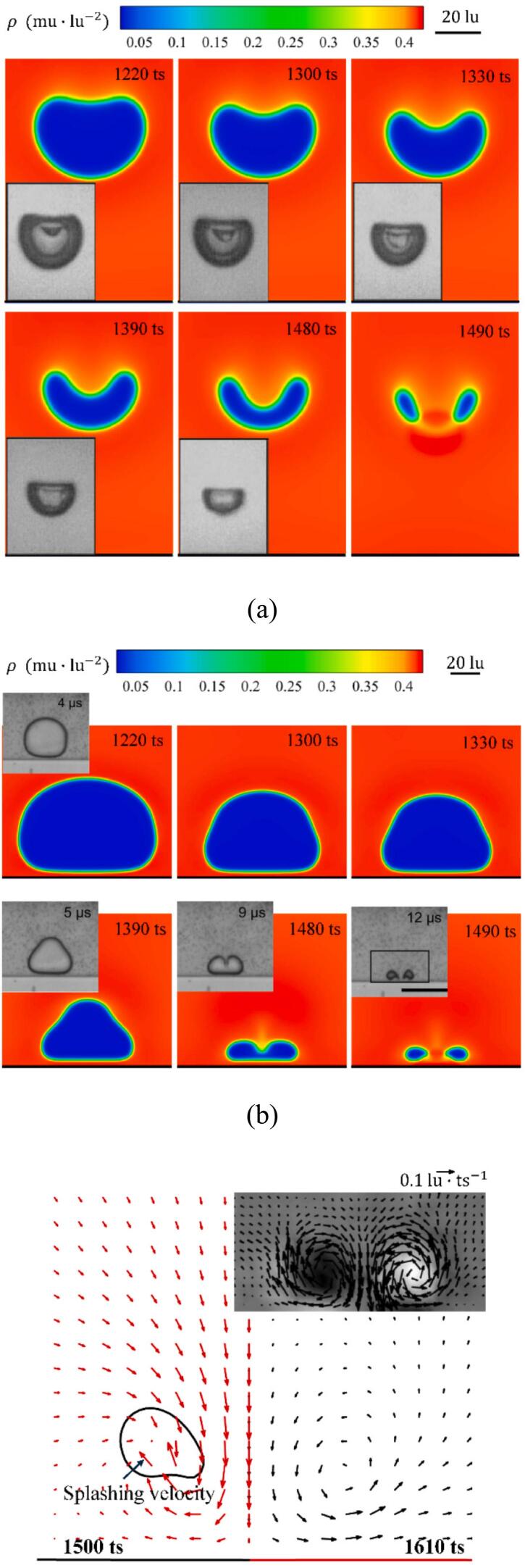
Snapshots showing (a) the final collapse stage of the cavitation bubble with γ=1.57 and y=90lu, , with inset images (front view) from Philipp et al. [57] at γ=1.6. (b) The final collapse stage with γ=0.81 and y=20lu, including insets (front view) from Zwaan et al. [58] at γ=0.8. (c) A snapshot of the “splashing” phenomenon at 1500 ts, and the vortex ring in the post-collapse stage (right panel, 1600ts), with an inset image from Zwaan et al. [58].

Furthermore, validation of the model is carried out by simulating the evolution of cavitation bubbles in the near-wall area, with the computational domain set to dimensions of $l_{x}=l_{y}=400lu$. A vapor nucleus with an initial hot spot of radius $r_{0}=5lu$ is positioned at y=20lu and 90lu, where y is the coordinate along the y-axis of the coordinate system. The lower boundary of the computational domain is set as a half-way bounce-back boundary, while the temperature boundaries around the computational domain are maintained at constant temperature boundaries. Note that, due to the unknown initial and boundary conditions in the experimental studies [57,58], particularly the initial inception temperature, only the qualitative comparisons are made here. The qualitative comparison between the simulation results and the experimental results is shown in Fig. 4. The bubble morphological evolution processes at y=20lu and 90lu are consistent with the experimental results for the same dimensionless bubble-wall distance γ. Especially for y=20lu, the splashing velocity caused by the collapse of the cavitation bubble in the final stage and the tangential vortices generated in the post-collapse stage are accurately reproduced, as depicted in Fig. 4 (c).

#### 2.3.3 Dual cavitation bubbles interact in the unbound region

Two cavitation bubbles interacting in an unbound domain is utilized for further validation. Two nuclei possessing an initial radius of $r_{0}=5\;lu$, are symmetrically placed within an 800lu×800lu computational domain. A constant temperature boundary condition of $T_{\infty }=0.5T_{c}$ is applied to all four boundaries, and the inception temperature within both nuclei are set as $T_{v}=T_{c}$.

For weak interaction, the evolution of the cavitation bubble radius can be expressed as [26]:

(16) $$\ln\left(\frac{r_{\infty }}{r}\right)\left({\dot{r}}^{2}+r\ddot{r}\right)-\frac{1-\left(\frac{r_{\infty }}{r}\right)}{2}{\dot{r}}^{2}=\frac{1}{\rho_{l}}\left(p_{v}-p_{\infty }-\frac{2\nu }{r}\dot{r}-\frac{\sigma }{r}\right)-\frac{r}{d_{\mathrm{ini}}}\left(2{\dot{r}}^{2}+r\ddot{r}\right)-\Omega \dot{r}\sqrt{t}$$

where $d_{\mathrm{ini}}$ is the starting distance between the centroids of the two bubbles. Furthermore, the film thinning thickness evolution process can be expressed as [21]:

(17) $$\frac{h(t)}{h_{0}}={\left(1-\frac{1}{2}\frac{{\dot{h}}_{0}}{h_{0}}\left(t-t_{0}\right)\right)}^{-2}$$

where h(t) represents the film thickness at time t, $h_{0}$ denotes the initial thickness of the liquid film, and ${\dot{h}}_{0}$ is the initial film thinning velocity.

**Fig. 5:**
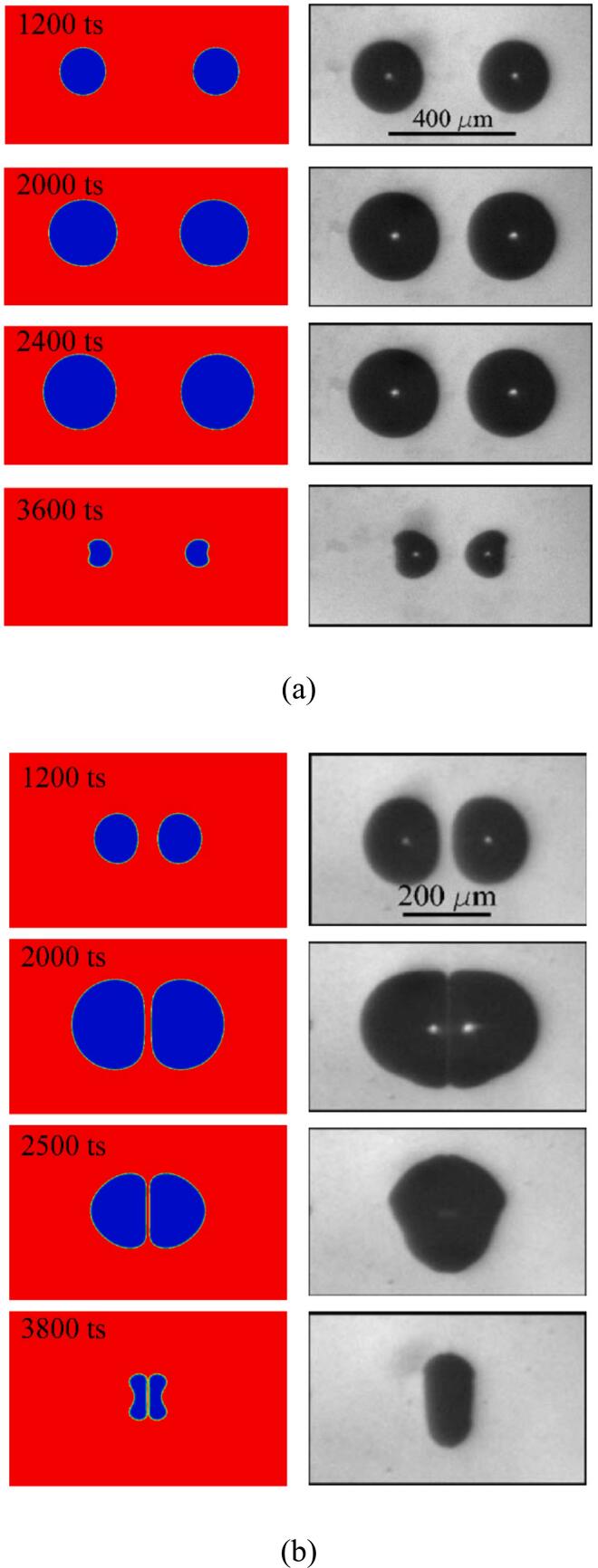
Snapshots of (a) the dual cavitation bubbles weak interaction mode with $d_{\mathrm{ini}}=300lu$ obtained by the present model (left panel), and compared to the experimental findings of Bremond et al. [59] (right panel). (b) The dual cavitation bubbles strong interaction mode with $d_{\mathrm{ini}}=100lu$ obtained by the present model (left panel), and compared to the experimental findings of Bremond et al. [59] (left panel).

**Fig. 6:**
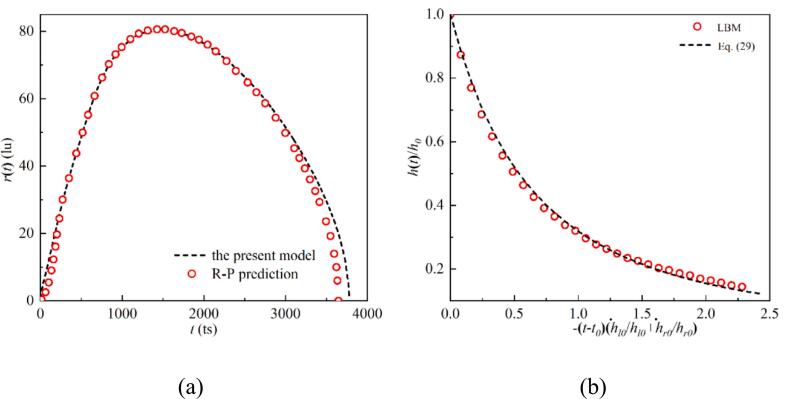
The evolution of (a) the bubbles’ radius for the weak interaction mode with $d_{\mathrm{ini}}=300lu$, and (b) the evolution of the liquid film thickness under strong interaction mode with $d_{\mathrm{ini}}=100lu$.

Fig. 5(a) shows the progression of the cavitation bubble morphology under the weak interaction regime and is compared with Bremond’s experimental result [59]. Note that the bubbles in Bremond’s study are attached-wall, with their centroids located on the wall. Therefore, the wall may be regarded as a symmetry wall, allowing t he two bubbles are regarded as if they are interacting in an infinite domain. The centerline connecting the cavitation bubble centroids is viewed as a pseudo-wall, with the bubbles eventually collapsing toward each other. The simulation results align with the experimental observations of bubble morphology evolution [59]. Fig. 6 (a) presents a quantitative comparison of the bubble radius with the modified R-P model [26]. During the growth phase, the bubble evolution closely follows the R-P theoretical solution. However, during the collapse stage, the bubbles lose their roundness, leading to a deviation between the simulation data and the R-P theory. The bubble morphology of the film thinning regime under the intense interaction between the two cavitation bubbles is depicted in Fig. 5(b). The simulation outcomes match the experimental findings from [59]. As the bubble grows, the inner gas–liquid interfaces gradually flatten, followed by their collapse toward each other. Fig. 6 (b) illustrates the liquid film evolution process, the simulation result agrees well with the theoretical solution.

## 3 Simulation results

### 3.1 Physical problems

**FIG. 7:**
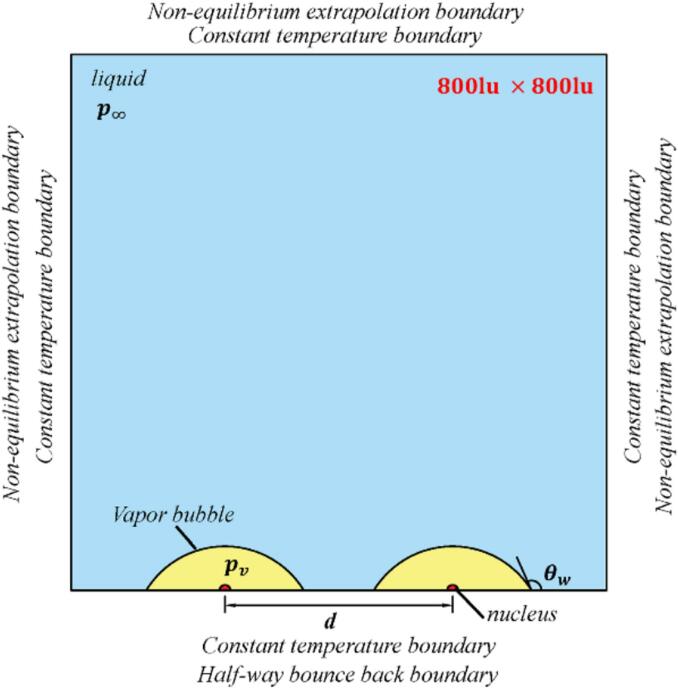
Computational domain scheme diagram of two attached-wall bubble interaction.

Fig. 7 displays the schematic of the computation domain with a size of 800lu×800lu. Two hemisphere nuclei with a distance d and radius r=5lu are initialized on the wall with

(18) $$\rho \left(x,y\right)=\frac{\rho_{l}+\rho_{v}}{2}+\frac{\rho_{l}-\rho_{v}}{2}\tanh\left\{\frac{2\left[\sqrt{{\left(x-x_{0}\right)}^{2}+{\left(y-y_{0}\right)}^{2}}-r_{0}\right]}{w}\right\}.$$

where ($x_{0}$, $y_{0}$) represents the initial centroid of the bubble, and w=5lu denotes the initialized thickness of the interface. The initial temperature is set as $0.5T_{c}$, with a liquid–vapor density $\rho_{g}/\rho_{v}\approx 720$. The remaining simulation parameters are consistent with those in section 2.3. Herein, four different equilibrium contact angle of the wall are chosen: $\theta_{w}=104^{{}^{\circ}}$ (hydrophobic wall), $90^{{}^{\circ}}$ (neutral wall), $68^{{}^{\circ}}$, and $37^{{}^{\circ}}$ (hydrophilic wall).

A constant temperature boundary is applied to all boundaries, with $T_{\infty }=0.5T_{c}$. The nonequilibrium extrapolation boundary condition is implemented on the top, left, and right boundaries. And the bottom boundary is set with a halfway bounce-back condition. Here, the constant temperature boundary is used to enforce that the wall and the boundary temperature tremains fixed at a prescribed value $T=0.5T_{c}$. The unknown temperature distribution functions at the boundary is obtained by the non-equilibrium extrapolation. Ensure with different $\theta_{w}$ s, the boundary temperature has no impact on the expansion and contraction rate of the cavitation bubble, and the evolution of the cavitation bubble mainly relies on changes in the boundary pressure.

To realize a attached-wall bubble with different dimensionless bubble–wall distance with the same bubble volume, the adhesion force is applied to achieve different wall wettability [60]:

(19) $$F_{ads}=-G_{w}\psi \left(x\right)\sum \omega_{i}S\left(x+e_{i}\right)e_{i}$$

where $S\left(x+e_{i}\right)=\phi \left(x\right)s\left(x+e_{i}\right)$, $s\left(x+e_{i}\right)$ is a switch function. s=0 for the solid nodes, s=1 for the fluid nodes, $\phi \left(x\right)$ is regarded as the pseudopotential ψ(x), $G_{w}$ is used to adjust the equilibrium contact angles of the wall.

Recently, Wang et al. [46] proposed a conversion method from lattice to physical units. It relies on the EOS parameters and the surface tension between the lattice system and the real fluid system. In this study, we use the vapor–water system at 293 K as the reference fluid. Table 1 presents the conversion parameters of the basic quantities [61]. The method proposed by Wen et al. is applied in this study to determine the exact contact angle, which is required to pinpoint the gas–liquid interface location. [62,63] Furthermore, the equivalent cavitation bubble radius R is introduced here, which is defined as $=\sqrt{V/\pi }$, where V is the volume of the cavitation bubble. The reference velocity is defined as $\mathrm{Uc}=\sqrt{2p_{\infty }/3\rho_{l}}$, where $p_{\infty }$ is the pressure when the boundary pressure curve stabilizes.

**Table 1:** Conversion parameters of the basic quantities, where $l_{c}$, $T_{c}$, $m_{c}$, and $t_{c}$ donte the length, temperature, mass, and time, respectively.

Basic quantities	$l_{c}$	$T_{c}$	$m_{c}$	$t_{c}$
Conversion parameters	$8.96\times 10^{-16}$	$2.7\times 10^{4}$	$1.14\times 10^{-27}$	$8.12\times 10^{-14}$

### 3.2 Bubble morphology evolution

According to Bremond et al., two bubbles interaction modes were distinguished according to their distance apart [59,64]. The interaction becomes weak when the initial distance between the two nuclei is greater than the sum of the maximum radii of the two bubbles. In contrast, the interaction mode is the strong interaction, which includes the film and coalescence modes.

#### 3.2.1 Weak interaction

**Fig. 8:**
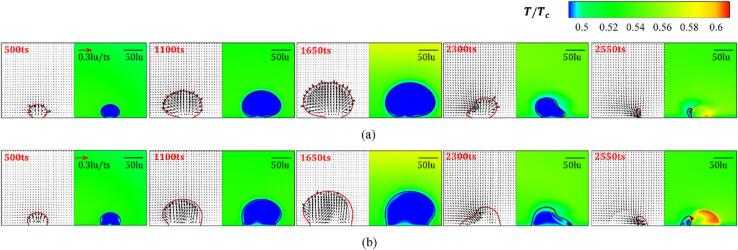
Variation of the velocity field (left panel) and temperature field (right panel) at different evolution stages for d=200lu with (a) $\theta_{w}=37^{{}^{\circ}}$ and (b) $\theta_{w}=104^{{}^{\circ}}$. The red and black solid lines denote the contour line $\rho =0.23mu∙{lu}^{-3}$.

The weak interaction intensity varies with different initial distance d ranges from 200 to 400lu is investigated. Fig. 8 presents the variation of the velocity and temperature fields in whole evolution periods for $\theta_{w}=37^{{}^{\circ}}$ and $104^{{}^{\circ}}$ with d=200lu. For t≤500ts, the nucleation and growth of the bubbles are impervious to each other, which resembles a single attached-wall bubble initial growth process. Since the phase transition from the liquid to the vapor occurs during the growth period and the latent heat for evaporation is supplied by the surrounding liquid, a low-temperature boundary layer forms near the interface of the liquid side. When the bubble radius increases, the liquid trapped between the two cavitation bubbles cannot be evacuated quickly. This, in combination with the contact point hysteresis effect, limits the lateral growth of inner bubble interface, which results in the attached cavitation bubbles undergoing asymmetric growth (t=1100ts). For the hydrophilic wall with $\theta_{w}=37^{{}^{\circ}}$, a high-temperature boundary layer forms on the vapor side, which stems from the continuous heating of the high-temperature liquid because of the insignificant expansion of the inner bubble wall. The cavitation bubbles achieve their maximum radius around 1650ts, due to the boundary pressure needing some time to transfer to the bubble walls. The wettability does not significantly influence the time when cavitation bubbles have their maximum radius.

Blake et al. suggested that the axis that symmetrically divides the line between the two nuclei can be considered a pseudo-wall, causing the bubbles to collapse toward one another [65]. Additionally, when the bubble achieves its maximum size and have γ>1, which results in the bubble collapses towards the wall. Owing to these two effects, an inclined re-entrant jet forms. For the hydrophobic wall, the bubble makes more extensive contact with the wall, which produces a larger curvature radius at the interface near the outer contact. The re-entrant jet can can infiltrate the cavitation bubble at large volumes for the hydrophobic wall. The temperature dramatically increases at the collapse stage due to the thermal energy released during the phase transition, and the resultant high-temperature liquid is transmitted outward with the pressure waves. The jet sweeps along the wall and causes the remaining parts to move toward each other. For the bubbles on the hydrophilic wall, the remaining parts finally depart from the wall due to the small contact area. Due to the jet’s splitting effect combined with the rapid contraction of the wall contact points, the region of elevated temperature created during the final collapse on the superhydrophobic interface becomes discontinuous, with high temperatures distributed on the top of the remaining cavitation bubble’s outer side and at the original contact points. For the hydrophilic wall, the microjet has a smaller angle with the solid wall, causing the cavitation bubble’s gas–liquid phase transition to mainly occur on the outer contact point, leading to a larger heated area due to the heat release from cavitation.

**Fig. 9:**
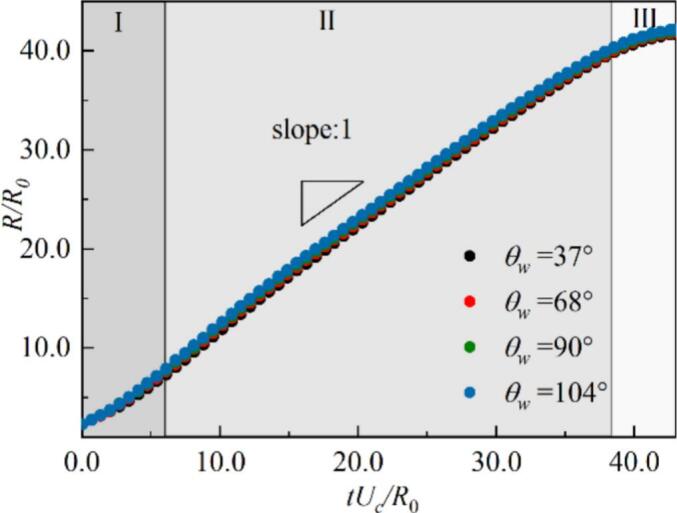
The evolution of non-dimensional equivalent bubble radius $R/R_{0}$ for different wall wettability in the growth stage.

**Fig. 10:**
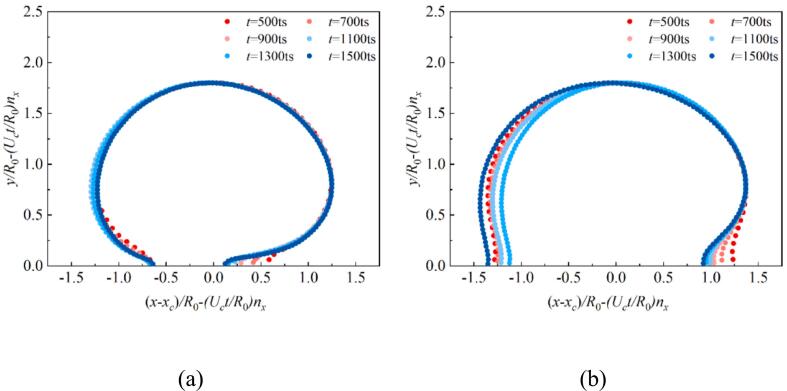
Comparison of the dimensionless interface shapes which re-scaled with the characteristic velocity $U_{c}t/R_{0}$ in the normal direction for (a) $\theta_{w}=37^{{}^{\circ}}$ and (b) $\theta_{w}=104^{{}^{\circ}}$, where $x_{c}$ is the bubble centroid at x direction.

Bremond et al. indicated that the approximate growth rate of the equivalent radius of a hemispherical attached-wall cavitation bubble can be expressed as $\dot{R}\;\sqrt{2\Delta p/3\rho_{l}}$ [59]. In The relationship between the normalized equivalent bubble radius $R/R_{0}$ and the dimensionless time $tU_{c}/R_{0}$ under different wettability in Fig. 9, where $R_{0}$ is the initial equivalent radius of the bubble nucleus, and $U_{c}=\sqrt{2p_{\infty }/3\rho_{l}}$. Here, $p_{\infty }$ denotes the boundary pressure after stabilization. For most of the time (Region II), the bubble radius grows linearly with a slope of approximately 1.0. The growth rate of the attached-wall cavitation bubble still follows the $\dot{R}\;\sqrt{2\Delta p/3\rho_{l}}$ law. Moreover, the evolution of the bubble radius under different wettability conditions almost overlaps, indicating that the influence of wettability on the radius evolution is limited. The growth of the attached-wall cavitation bubble is dominated by inertial forces, which is consistent with the findings of Bremond et al. [59] and Saini [66]. Fig. 10 illustrates the evolution of the non-dimensional cavitation bubble shapes at $\theta_{w}=37^{{}^{\circ}}$ and $104^{{}^{\circ}}$, with the shapes rescaled by the non-dimensional length $tU_{c}/R_{0}$ ​. The outer interface of the cavitation bubbles exhibits self-similarity under both wettability conditions, with the primary differences occurring near the contact points in the initial growth stage, caused by the adjustment of the initial contact angle. Note that, between 1100ts and 1300ts​, the outer interfaces of the bubble at different times nearly overlap, corresponding to the linear region II in Fig. 10. However, the evolution of the inner interface is affected by the adjacent bubble, with its growth is no longer solely governed by inertial. This leads to noticeable differences in the interface shape, which are particularly pronounced with $\theta_{w}=104^{{}^{\circ}}$.

**Fig. 11:**
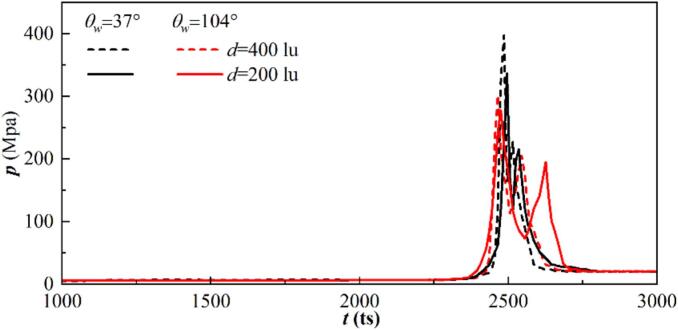
Variation of the peak pressure in the computational domain for varying initial distances d and wall wettability for the weak interaction mode.

Fig. 11 illustrates the progression of peak pressure values within the computational domain for the initial distances d=200 and 400lu on the hydrophilic and hydrophobic walls. Two peaks are observed in the final collapse stage, corresponding to the re-entrant jet penetrating the cavitation bubble and the collapse of the remaining parts. As indicated in our previous study [45], increased contact area and curvature radius lead to earlier breakdown of the cavitation bubbles on the hydrophobic wall under a larger volume. Since the volume of the remaining part is large, more time is needed for the final collapse, which results in an extended duration interval between the two peaks for the hydrophobic wall compared to that for the hydrophilic wall. For d=400lu and $\theta_{w}=37^{{}^{\circ}}$, the maximum collapse pressure is 397.4 MPa, which is 1.32 times that for $\theta_{w}=104^{{}^{\circ}}$. Furthermore, the collapse intensity decreases with increasing initial distance d. The interaction intensity between two bubbles increases with decreasing d, and the second Bjerknes force stemming from the neighboring bubble also increases, which amplifies the tendency of the cavitation bubble to migrate toward the central axis. As the coupling effect decreases, the intersection angle between the re-entrant jets and the wall increases, while the cavitation bubble is punctured in a smaller volume, resulting in an increase of the collapse time. For $\theta_{w}=37^{{}^{\circ}}$ and d=400lu, the first collapse pressure is 336.54MPa, which is 1.21 times that for d=200lu with the same wettability.

#### 3.2.2 Strong interaction

**Fig. 12:**
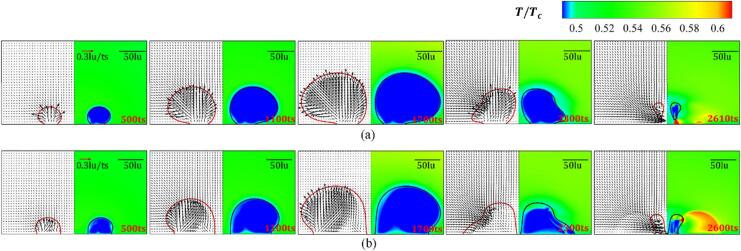
Variation of the velocity field (left panel) and temperature field (right panel) at different evolution stages for d=90lu with (a) $\theta_{w}=37^{{}^{\circ}}$ and (b) $\theta_{w}=104^{{}^{\circ}}$. The red and black solid lines denote the density contour line with $\rho =0.23mu∙{lu}^{-3}$.

The film thinning mode implies that the interaction dynamics change to the strong interaction mode. Cavitation bubbles initially nucleate and grow under the influence of the evolution of the neighboring cavitation bubble (t<500ts), and the expansion rate of the outer interface of the cavitation bubbles is larger than that of the inner interface, as depicted in Fig. 12. Due to the high-temperature fluid heating, a boundary layer on the vapor side with high-temperature of the inner wall is afforded at t=500ts. The boundary layer becomes more obvious as the wall shifts from hydrophilic to hydrophobic. For the hydrophilic wall, the flattened surface of the cavitation bubbles is not as remarkable after the cavitation bubble expands to its largest volume (t=1650ts). In contrast, a thin layer of liquid forms between the inner surfaces of the two bubbles on the hydrophobic wall, due to the enlargement of the contact area. However, the combined pressure difference and the unbalanced Young’s force are not sufficient for overcoming the surface tension of the liquid film. The inclined re-entrant jets are afforded because of the combined influence of adjacent cavitation bubble and the wall. The cavitation bubble collapse results in more pronounced re-entrant jets that are parallel to the wall, resulting in the detachment of the remaining part from the hydrophilic wall. The phase transition between the vapor and liquid in the cavitation bubble occurs mainly near the wall, with high temperatures distributed close to the solid wall. For the hydrophobic wall, the inner contact point of the remaining part almost overlaps due to the sweeping effect. Due to the limited movement range of the cavitation bubble’s contact point during the sweeping process on the hydrophobic wall, the gas–liquid phase transition occurs more uniformly on the outer interface, forming a continuous and uniform high-temperature region.

**Fig. 13:**
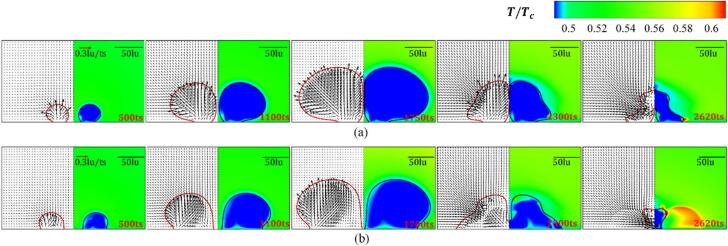
Variation of the velocity field (left panel) and temperature field (right panel) at different evolution stages with (a) d=60lu and $\theta_{w}=37^{{}^{\circ}}$, and (b) d=80lu and $\theta_{w}=104^{{}^{\circ}}$. The red and black solid lines denote the density contour line with $\rho =0.23mu∙{lu}^{-3}$.

**Fig. 14:**
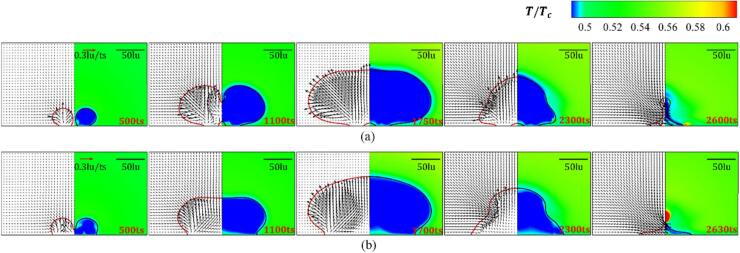
Variation of the velocity field (left panel) and temperature field (right panel) at different evolution stages for d=30lu with (a) $\theta_{w}=37^{{}^{\circ}}$, and (b) $\theta_{w}=104^{{}^{\circ}}$. The red and black solid lines denote the density contour line with $\rho =0.23mu∙{lu}^{-3}$.

This study reports two merging modes: merging in the collapse period (Fig. 13) and merging in the growth period (Fig. 14). The cavitation bubble merging mode changes as the wall varies from hydrophilic to hydrophobic. The velocity distribution and bubble morphology during the merging in the collapse period of two cavitation bubbles on the hydrophilic wall are presented in Fig. 13 (a). The liquid layer between two bubbles gradually becomes thinner after the liquid between two bubbles is squeezed out, and the inner walls come into contact. Bubble coalescence occurs when the hydrodynamics is energetically strong enough to overcome the surface tension on the liquid film (t=2300ts). Before coalescence occurs, the cavitation bubbles are influenced both by the adjacent cavitation bubble and the wall. Moreover, inclined re-entrant jets are formed, and the coalescence of the two bubbles little influences the re-entrant jet characteristics. Since a two-dimensional model is considered herein, the liquid between the inner contact points and coalescence point is not discharged in time, which affects the subsequent bubble morphology and hydrodynamics evolution.

For the hydrophobic wall, the inner contact points of the two cavitation bubbles first contact each other due to the outward expansion during the collapse period. Since the dynamic contact angle is greater than $\theta_{w}$, the unbalanced Young’s forces point outward of the cavitation bubbles and parallel to the wall, resulting in momentum redistribution. Therefore, the coalescence point is first reported at the inner contact points for the hydrophobic wall (Fig. 13 (b), t=2300ts).

Fig. 14 illustrates another coalescence mode for two attached-wall cavitation bubbles, with coalescence in the growth stage. The interaction dynamics are similar to those under bubble coalescence during the collapse stage. For the hydrophilic wall, the coalescence happens at the contact point of the middle inner bubble wall (t=1100ts). Furthermore, for the hydrophobic wall, the coalescence point is on the contact point (t=500ts). After the coalescence, the cavitation bubble continuously expands in the lateral direction. Affected by the adjustment of the interfacial deformation, the vertical expansion velocity of merging bubble is inhibited, and finally, an elliptical capsule-like cavitation bubble forms at its maximum volume. Assuming the surface tension remains constant during the bubble expansion and initial collapse period. According to the Laplace law, Δp=σ/R, the larger radius of curvature at the apex of the cavitation bubble causes a smaller pressure difference. In contrast, the pressure difference in the lateral direction of the bubble wall is larger. This results in the compression of the merged bubble in the lateral direction. This, combined with the wall effects, causes the creation of angled re-entrant jets. Since the compression rate is greater in the lateral direction, the bubble is punctured first by the horizontal component of the jets, and a short interval of about 20ts is reported between the two collapses.

**Fig. 15:**
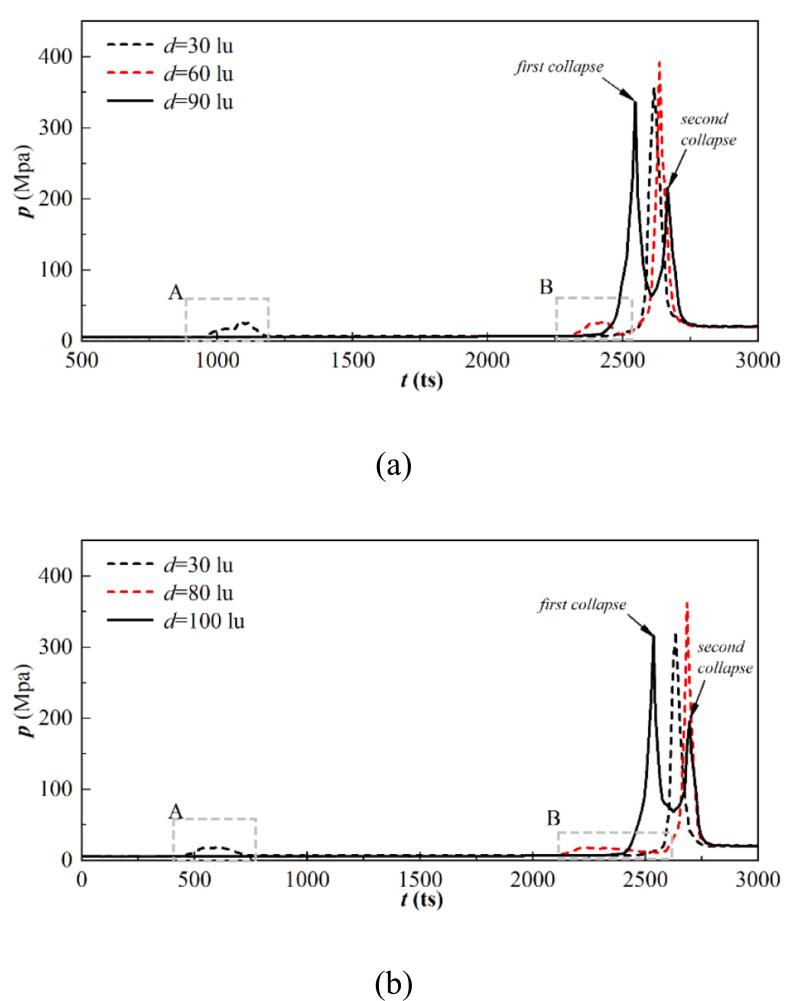
Variation of the peak pressure in the computational domain for varying initial distance d and (a) hydrophilic wall with $\theta_{w}=37^{{}^{\circ}}$, and (b) hydrophobic wall with $\theta_{w}=104^{{}^{\circ}}$ for the strong interaction mode.

The variation of the peak pressure at each timestep with the initial distance and wall wetting conditions are shown in Fig. 15. As the bubble coalescence in the collapse stage, time gap between two collapses is short and the curves comprise only one peak. The energy required for the nucleation of a hole in the film is approximately $\sigma h^{2}$, and the activation energy correlates with the temperature of the local film. The increase of the local temperature results in a high local pressure; thus, a pressure fluctuation is observed in the curves for the coalescence modes, as depicted in the A and B regions in Fig. 15. For the film thinning mode, two peaks are observed as the weak interaction mode. For the hydrophilic wall with $\theta_{w}=37^{{}^{\circ}}$, the time interval between the two peaks is 120ts, which is slightly less than that for the hydrophobic wall with $\theta_{w}=104^{{}^{\circ}}$, which is about 160ts. Moreover, the film thinning collapse intensity is smaller than that of the coalescence mode. For the film thinning mode, owing to the large time interval between the two collapses, the bubble energy is not released within a brief timeframe. The high pressure generated by the bubble being punctured seems larger than that generated by the collapse of the remaining parts. For the coalescence mode, even the interface fusion and deformation need to overcome the viscous and inertial forces, and the two collapses occur in short periods, resulting in a more concentrated energy release process and a higher collapse intensity. For hydrophilic wall with $\theta_{w}=37^{{}^{\circ}}$, the maximum collapse pressure at d=60lu is 392.04MPa, which is 1.16 times of that at d=90lu. For the hydrophobic wall with $\theta_{w}=104^{{}^{\circ}}$, the maximum collapse pressure at d=60lu is 362.04MPa, which is 1.14 times of that at d=90lu.

### 3.3 Heat flux characteristics

The impact of wall wettability on heat transfer intensity between the wall and the fluid is assessed by introducing a dimensionless temperature, which is defined as [45]

(20) $$\eta =\frac{T(y)}{T_{w}}$$

Herein, the temperature at y=5lu is selected to reduce the impact of the heat diffusion calculation. For η>1, heat transfer occurs from the liquid to the wall, vice versa.

**Fig. 16:**
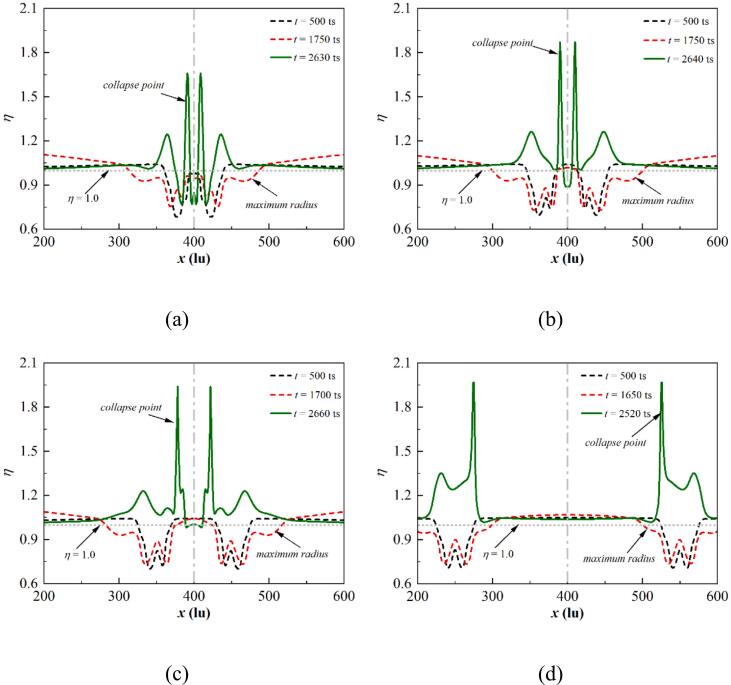
Dimensionless temperature distribution at different stages for $\theta_{w}=37^{{}^{\circ}}$ with (a) d=30lu, (b) d=60lu, (c) d=100lu, and (d) d=300lu, where x=400lu is the bubble central axis. The bubble central axis is marked by the gray dash-dot line, while the gray dotted line illustrates η=1.

Fig. 16 illustrates the dimensionless temperature distribution at different stages for $\theta_{w}=37^{{}^{\circ}}$ under different initial d. For d=30lu, the bubble coalescence occurs at t=500ts. The dimensionless temperature distribution is W-shaped. The minimum dimensionless temperature is reported at the points where the outer bubble wall makes contact, which arises from the local dramatic phase change, causing heat absorption to occur on the interface. Moreover, the vapor near the wall is heated by the wall in the bubble center, and a local high-temperature boundary forms. A peak is observed on the central axis with η close to 1. For the cases without bubble coalescence at t=500ts, two typical W shapes are observed. Due to the different growth and phase change rates at each contact point, the growth rate of the inner contact point is smaller than the outer interfaces. Consequently, the minimum η value observed at the contact point close to the central axis is smaller than that observed at the outer interface. The impact of the adjacent cavitation bubble diminishes as d increases, resulting in the decrease of the expansion rate difference between two contact points of the cavitation bubble. Thus, the difference of the η value at the contact points of one bubble also decreases.

When the bubble reaches its maximum at t=1750ts for d=30lu, the η distribution shaper still roughly exhibits a W shape. Since the liquid between two bubbles is not discharged in time, an unphysical phase change is observed on the interface between the bubble and the undischarged liquid, resulting in the oscillation of the η curve in the bubble. This unphysical phenomenon will be addressed in subsequent three-dimensional simulations. When the bubble attains its largest volume with d>30lu, the η curve has a distribution similar to that at t=500ts. A low-temperature boundary layer in the liquid phase is reported due to the latent heat supply. Furthermore, the boundary high pressure transfers to the liquid in the computational domain, resulting in a rise in the dimensionless temperature of the liquid phase with η>1. As the pressure gradient across the cavitation bubble decreases, the phase change intensity weakens, and the minimum η value is larger than that at t=500ts.

The bubbles are ultimately ruptured by two jets acting in opposite directions. The high pressure arising from the asymmetric deformation of the cavitation bubbles leads to the acceleration of the phase change rate, yielding two peaks symmetric to the central axis. The maximum η value first increases and then decreases with increasing d due to the exothermic superposition effect. Moreover, a set of symmetric peaks is located outside the highest peaks, which stem from the increased pressure at the contact points resulting from the concentration of momentum. A swift inward shrinking of the outer contact points occurs during the final collapse phase, which accelerates the local phase change rate.

**Fig. 17:**
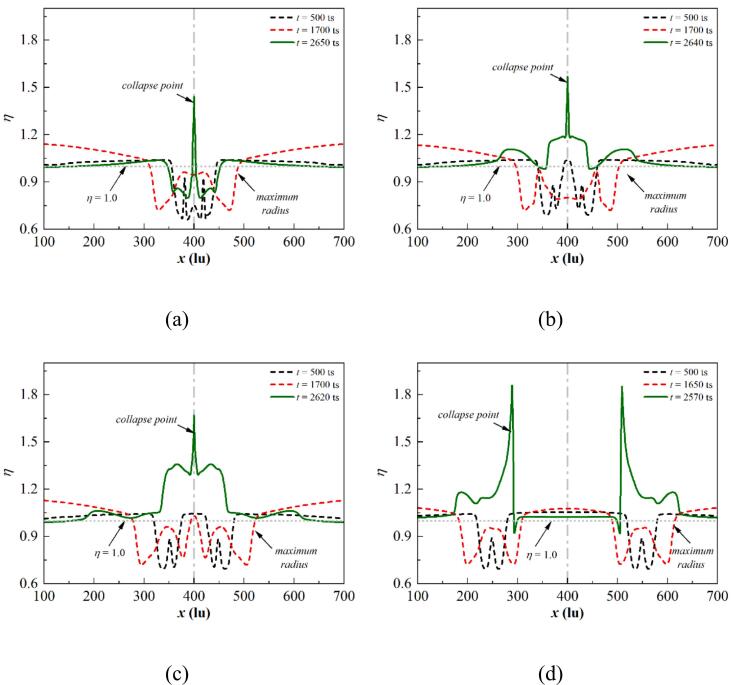
Dimensionless temperature distribution at different stages for $\theta_{w}=104^{{}^{\circ}}$ with (a) d=30lu, (b) d=60lu, (c) d=100lu, and (d) d=300lu, where x=400lu is the bubble central axis. The bubble central axis is marked by the gray dash-dot line, while the gray dotted line illustrates η=1.

Fig. 17 depicts the dimensionless temperature distribution at different stages for $\theta_{w}=104^{{}^{\circ}}$ under different initial d. For d=30lu, three peaks are observed in the η distribution curves at t=500ts inside the bubble. The symmetrically distributed peaks on both sides arise from the formation of a high-temperature boundary layer due to wall heating, while the peaks on the symmetry axis correspond to exothermic heating due to interface coalescence. For d>30lu, the distribution of η at t=500ts is similar to that for $\theta_{w}$ = 37° with two typical W-shaped curves.

The bubble contact radius increases as the wall changes from hydrophilic to hydrophobic, resulting in a wider spread of the η distribution in the W-shaped mode at t=1700ts under the same d. Note that the η value on the central axis is close to 1 at t=30lu. Since the two bubbles are too close to each other, the central axis stays in the high-temperature boundary layer due to the continuous heating of the wall. Moreover, the merging of two bubbles in the growth period is not influenced by the boundary layer heated due to wall heating on the central axis; consequently, the η value on the central axis is close to 0.8 for d=60lu. For d=100 and 300lu, bubble coalescence no longer occurs, and the high-temperature liquid exists in the gap between the two bubbles; subsequently, the η value on the central axis is close to 1.

At the collapse point, only one peak is reported for d=30, 60, and 100lu. The bubble is pierced by two opposing jets at d=30lu (Fig. 14 (b)), and a high-temperature region that is symmetrically distributed along the central axis forms above the bubble. The remaining parts prevent the high-temperature fluid sweeps along the wall, and the η distribution remains in a W shape. For d=60 and 100lu, the bubbles are pierced, triggering the first collapse, forming high-temperature liquid that propagates outward with the pressure wave, resulting in the formation of high-temperature regions on both sides of the peak. The jets sweep along the wall and prompt the remaining portion to collapse, leading to the η peak forms on the central axis. As the distance increases to d=300lu, double peaks are observed on the η distribution curve at t=2570ts, which is similar to that for $\theta_{w}=37^{{}^{\circ}}$.

## 4 Discussion

**Fig. 18:**
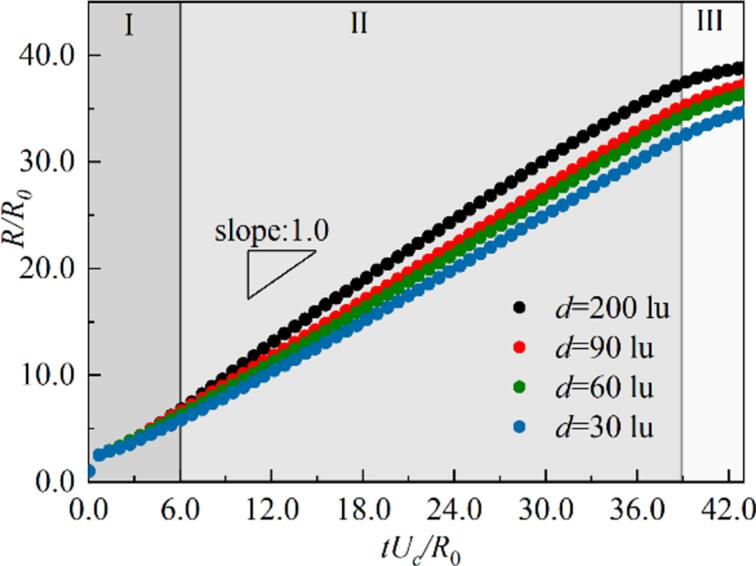
The evolution of non-dimensional equivalent bubble radius $R/R_{0}$ for different d s in the growth stage.

The interaction strength of neighboring cavitation bubbles directly affects the growth rate of the cavitation bubble equivalent radius. Fig. 18 represents the evolution of the bubble radius for different d s with $\theta_{w}=37^{{}^{\circ}}$. The slope of the dimensionless radius evolution increases from 0.80 to 0.97 as d increases, approaching the asymptotic growth velocity $U_{c}$ of a hemispherical cavitation bubble obtained by Bremond et al. [59]. In cases where no coalescence happens during growth stage, the flow field induced by neighboring bubbles causes the bubble interface to gradually deformed/ flatten. The energy required for interface deformation increases, leading to a reduction in the bubble’s growth rate. In scenarios where bubble coalescence occurs during growth, the thermal effects and interface deformation caused by the coalescence further reduce the growth rate, resulting in an evolution slope of less than 1. Additionally, despite different interaction modes, the radius evolution still follows a linear trend, indicating that the process remains inertia-dominated throughout.

**Fig. 19:**
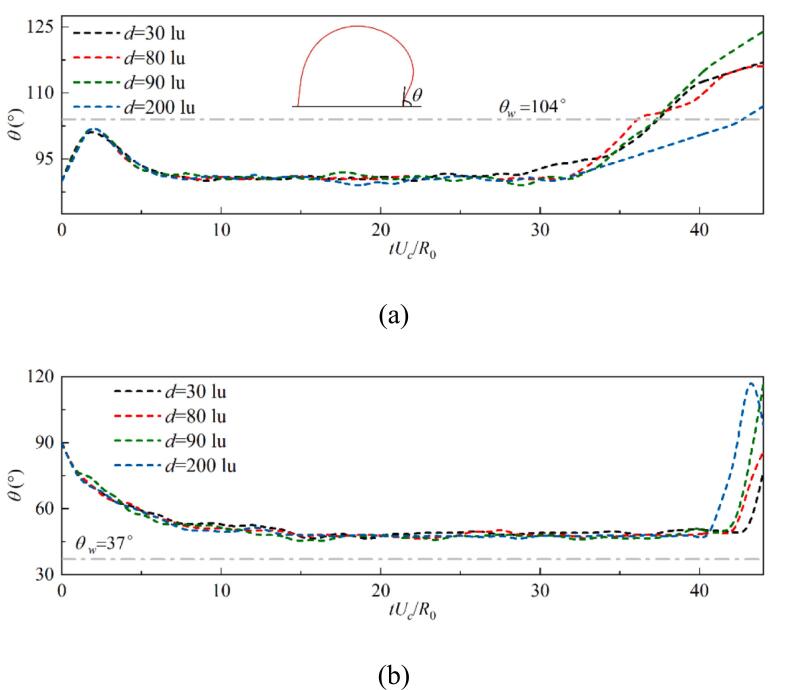
The evolution of dynamic contact angle for (a) $\theta_{w}=104^{{}^{\circ}}$, the inset shows a schematic of the dynamic contact angle on the outer side of the cavitation bubble, and (b) $\theta_{w}=37^{{}^{\circ}}$.

The effects of different interaction modes on the dynamic behavior of the contact points on the outer side of the cavitation bubble is analyzed, including the evolution of the contact angle and the contact point velocity during the growth stage. The dynamic contact angle evolution under different wettability conditions is presented in Fig. 19 with $\theta_{w}=104^{{}^{\circ}}$ and $37^{{}^{\circ}}$. For $\theta_{w}=104^{{}^{\circ}}$, the dynamic contact angle of the cavitation bubble rapidly increases towards the equilibrium contact angle after an initial adjustment under the influence of the unbalanced Young’s force. Under the influence of the bubble interface deformation and no-slip boundary, the contact angle decreases to about $90^{{}^{\circ}}$. The dynamic contact angle reaches a stable stage where the bubble profile reaches a self-similar stage. Regardless of the different interaction modes, the outer dynamic contact angle follows a consistent evolution during the growth stage and the early collapse stage. This indicates that the effects of heat release and interface morphology changes caused by deformation and coalescence of the bubble along the inner wall under strong interaction remain localized. This dynamic angle evolution behavior at $\theta_{w}=37^{{}^{\circ}}$ is similar to that of $\theta_{w}=104^{{}^{\circ}}$, except that the contact angle in the stable stage is approximately $50^{{}^{\circ}}$, which is larger than $\theta_{w}$. Differences in the dynamic contact angle evolution become noticeable during the final collapse phase, attributed to variations in the interaction strength between bubbles. These differences affect the angle of the resulting micro-jets, leading to distinct interface deformations.

**Fig. 20:**
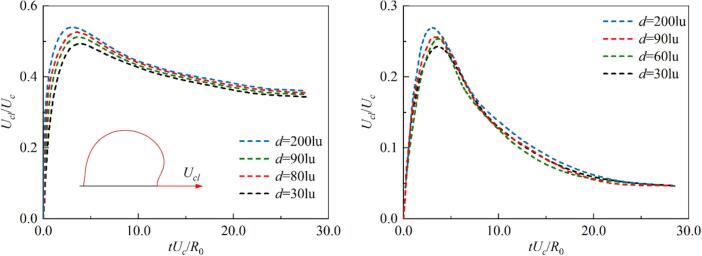
The evolution of dimensionless contact point velocity for (a) $\theta_{w}=104^{{}^{\circ}}$, the inset shows a schematic of the contact point velocity on the outer side of the cavitation bubble, and (b) $\theta_{w}=37^{{}^{\circ}}$.

**Fig. 21:**
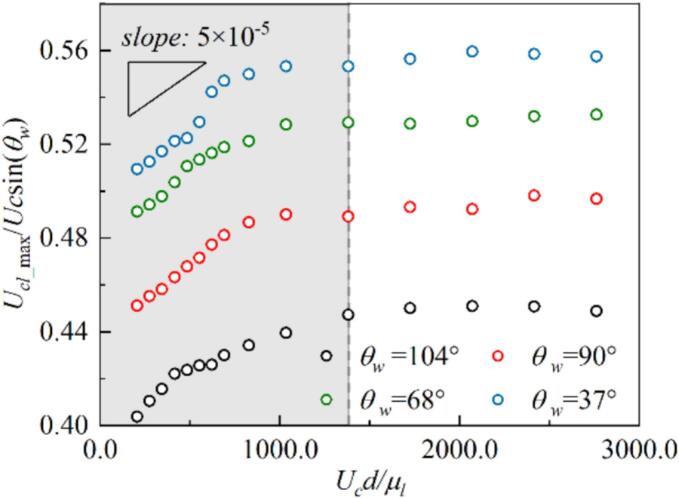
The relationship between the maximum contact point velocity $U_{cl\_max}$, the contact angle $\theta_{w}$ as well as the initial spacing between bubbles d during the growth stage.

Fig. 20 shows the evolution of the dimensionless velocity of the outer contact point of the cavitation bubble during the growth phase under different initial interval distances *d*. In the initial stage, the contact point velocity for $\theta_{w}=104^{{}^{\circ}}$ and $37^{{}^{\circ}}$ increases to a peak value under the combined influence of the unbalanced Young’s force and the pressure difference, and then decays to a stable value. The interaction strength between bubbles increases as the initial distance d decreases, and the compression from neighboring bubbles pushes the bubble centroid to move outward. However, the $U_{\mathrm{cl}}$ still increases with d. This indicates that the pressure difference is the dominant factor governing the contact point velocity. After the cavitation bubble reaches a stable shape, the outer contact point velocity converges due to viscous effects, following $u\propto e^{-\nu t}$ [66]. Furthermore, the unbalanced Young’s force induced by the dynamic contact angle acts in opposite directions for $\theta_{w}=104^{{}^{\circ}}$ and $37^{{}^{\circ}}$. This leads to the maximum velocity of the outer contact point for $\theta_{w}=104^{{}^{\circ}}$ being approximately 1.96 to 2 times that of $\theta_{w}=37^{{}^{\circ}}$, while the final stable slip velocity is about 4 times that of $\theta_{w}=37^{{}^{\circ}}$. Fig. 21 illustrates the relationship between the maximum contact point velocity $U_{cl\_max}$ and the contact angle $\theta_{w}$ as well as the initial spacing between bubbles d during the growth stage. Two dimensionless parameters are defined here: the y-axis represents the product $\theta_{w}$ and $U_{cl\_max}$, while the x-axis shows the dimensionless Reynolds number $Re=U_{c}d/\mu_{l}$. In the range where Re≤1000, the contact line velocity $U_{cl\_max}$ almost increases linearly with Re with a slope $5\times 10^{-5}$. However, the value gradually enters a stable value with Re≳1300.

**Fig. 22:**
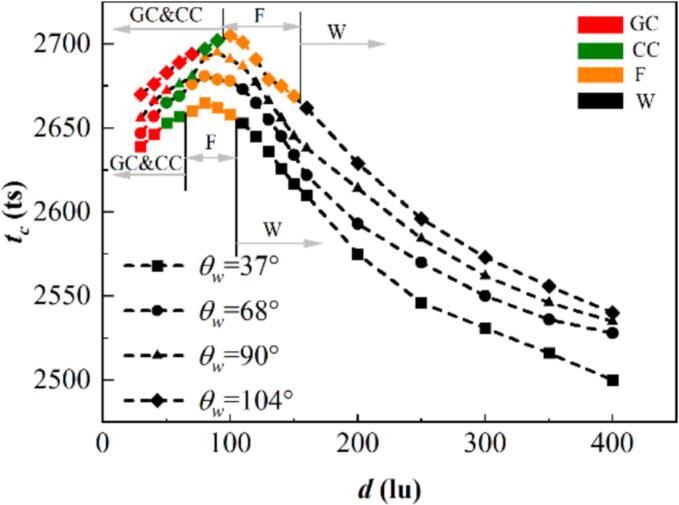
Bubble lifetime $t_{c}$ and the interaction modes with different initial distance d and wall wettability $\theta_{w}$, where GC denotes the coalescence in the expansion period, CC indicates the coalescence in the shrinkage period, and F and W denote the film thinning and weak interaction modes, respectively.

Earlier studies indicated that the interaction mode transition in the infinite region is related to the initial distance, boundary pressure, and surface tension [64]. The present study also indicates that the transition of attached-wall bubbles is related to the wall wettability. Fig. 22 presents the bubble lifetime $t_{c}$ and the interaction modes vary with the initial distance d and wall wettability $\theta_{w}$. The results indicate that the lifetime of cavitation bubbles decreases as the wall shifts from being hydrophobic to hydrophilic with the same initial distance d, which is because bubbles on hydrophilic walls collapse earlier with a smaller intensity than those on hydrophobic walls. Thus, smaller pressure and jets velocity act on the remaining parts, causing the remaining parts to have a large volume and a small compress rate. Under the coalescence mode, the bubble lifetime increases with the initial distance d. During the coalescence process during the bubble expansion period, the pressure work is utilized for the bubble interface deformation, resulting in a smaller coalescence cavitation bubble and a shorter lifetime. Moreover, the coupling effect between two bubbles weakens with increasing d for the non-coalescence mode. The high-pressure fluid is more likely to enter the gap between the two cavitation bubbles to fill the low-pressure region formed by the bubbles contract with increasing d, reducing the pressure difference between the two sides of the cavitation bubbles, which results in the shortening of the bubble lifetime.

Additionally, when the wall changes from hydrophilic to hydrophobic, the interval of the strong interaction mode and the coalescence interval decrease. This is attributed to the different coalescence ways with different wall wettability. For the hydrophobic wall, the contact radius of the bubbles increases during the bubble expansion period, and after the contact points of the two cavitation bubbles overlap, coalescence is more likely to occur due to the unbalanced Young’s force. For the hydrophilic wall, the coalescence process of the bubble inner wall necessitates the formation of a hole on the liquid layer, which requires more energy.

**Fig. 23:**
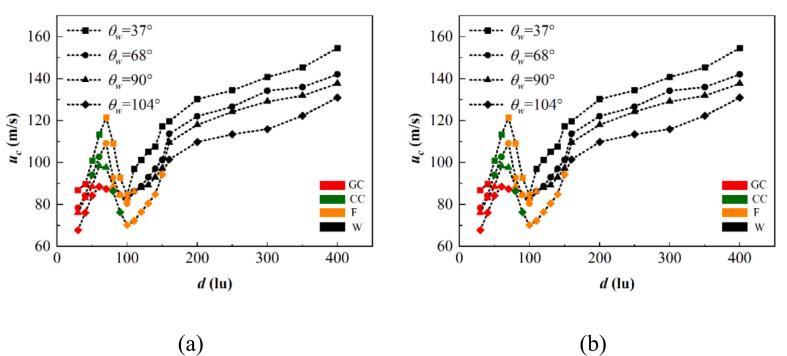
(a) The highest jet velocity and (b) the highest dimensionless temperature vary with initial distance d and wall wettability $\theta_{w}$, where GC denotes bubbles merging in the growth period, CC indicates bubbles merging in the shrinkage period, and F and W denote the film thinning and weak interaction modes, respectively.

Fig. 23 represents the peak jet velocity and temperature with different initial distance d and $\theta_{w}$. Under the same initial distance, the bubble collapse intensity increases for the wall transitions from hydrophobic to hydrophilic. The variation of the maximum re-entrant jet velocity is dominated by the initial distance d; the velocity first increases, then decreases, and finally increases, reaching the first peak at d=6070lu. For the hydrophilic wall cases, the initial peak occurs in the early stage of the film thinning mode (d=70lu). The energy consumption stemming from the coalescence of the bubble walls results in a lower collapse intensity in the coalescence mode compared to that in the film thinning mode. For the hydrophobic wall, i.e., $\theta_{w}=104^{{}^{\circ}}$, the coalescence of the cavitation bubbles commences from the contact point, resulting in less energy consumption during the coalescence process compared to that for the hydrophilic wall, and the first peak appears at d=60lu and is located in the coalescence mode. In the film thinning mode, the interaction intensity between two bubbles decreases with increasing d, diminishing the splashing effect caused by two opposing re-entrant jets.

As the interaction behaviors between the two bubbles transform from film thinning to weak interaction mode with increasing d, the distance between the cavitation bubble and the pseudo-wall increases. Consequently, the cavitation bubble lifetime decreases, which causes the bubble to release energy in a short time and increases the collapse intensity. This is consistent with Brujan et al. [67], who indicated that the bubble collapse intensity increases with the dimensionless bubble–wall distance.

The change in the highest collapse temperature is similar to the velocity variation trend: increase, decrease, and then increase. The initial growth in the coalescence mode and the first peak in the film thinning mode are attributed to the exothermic superposition effect of two bubbles. Since the film thinning mode on the hydrophobic wall occurs at large d, the first peak appears at a large d. Then, as the separation between two bubbles widens, the exothermic superposition effect decreases, resulting in a reduction of the maximum temperature as d increases. Finally, as the interaction mode changes from the film thinning to the weak interaction mode, the collapse intensity strengthens with larger d and the temperature undergoes a growth stage.

## 5 Conclusions

In this study, a nonorthogonal hybrid thermal LB cavitation model is proposed to investigate the interaction of two attached-wall cavitation bubbles. The flow and density fields are obtained using a nonorthogonal pseudopotential model, while the temperature distribution is solved via the fourth-order Runge–Kutta method. The study systematically examines weak interaction, film thinning, and coalescence, revealing distinct bubble morphologies and flow characteristics during collapse.

Wall wettability significantly influences coalescence dynamics. On the hydrophilic wall, coalescence initiates at the inner interfaces, nucleating a hole in the liquid film with associated exothermic heat release and pressure fluctuations. On the hydrophobic wall, coalescence occurs due to the overlap of inner contact points and unbalanced Young’s force, requiring less energy and resulting in smaller pressure fluctuations. The expansion of bubbles on the hydrophobic wall leads to a wider interaction range under strong interactions.

Opposing re-entrant jets consistently appear during bubble interactions. Inclined jets form due to interactions with both the wall and adjacent bubbles. In the growth stage coalescence mode, a small jet-wall intersection angle leads to vertical puncturing of bubbles. As d increases, bubbles are first pierced by inclined jets, followed by microjets sweeping along the wall, either detaching the bubbles (hydrophobic wall) or pushing them toward the central axis (hydrophilic wall). A larger piercing volume on the hydrophobic wall results in lower collapse intensity. The time interval between the first collapse and the collapse of remaining parts increases with *d*, producing two peaks in the pressure curve for larger *d*.

Heat flux characteristics show that the maximum dimensionless temperature first rises and then declines with increasing d due to exothermic superposition effects under strong interactions. On hydrophilic walls, subsequent collapse results in a single-peaked temperature distribution, whereas on hydrophobic walls, departing bubbles create a double-peak distribution. For weak interactions, bubble growth remains inertia-dominated, following $U\propto \sqrt{2p_{\infty }/3\rho_{l}}.$ In strong interactions, growth slows but remains linear, with the outer bubble shape exhibiting self-similarity across interaction modes. The dynamic contact angle evolution is largely unaffected, but the outer contact point velocity increases with d before stabilizing.

However, in the case of two cavitation bubbles coalescing on a hydrophobic wall, the liquid phase between the bubbles was not discharged in time, a limitation attributed to the 2D model used in this study. This resulted in some unrealistic distributions of the instantaneous heat flux characteristics, and the evolution process of the interface during coalescence could not be accurately captured. This will be solved using a 3D model [68] in our future studies.

## CRediT authorship contribution statement

**Weidong Gan:** Writing – original draft, Methodology, Investigation. **Shicheng Li:** Writing – review & editing, Writing – original draft, Supervision, Methodology, Data curation. **Xiaolong He:** Writing – review & editing, Writing – original draft, Supervision, Methodology, Investigation, Funding acquisition, Data curation. **Dianguang Ma:** Data curation.

## Declaration of competing interest

The authors declare that they have no known competing financial interests or personal relationships that could have appeared to influence the work reported in this paper.

## References

[1] Huang B., Wu Q., Wang G. (2014). Numerical investigation of cavitating flow in liquid hydrogen. Int. J. Hydrogen Energy.

[2] Le A.D., Okajima J., Iga Y. (2019). Numerical simulation study of cavitation in liquefied hydrogen. Cryogenics.

[3] Zhang S., Li X., Hu B., Liu Y., Zhu Z. (2019). Numerical investigation of attached cavitating flow in thermo-sensitive fluid with special emphasis on thermal effect and shedding dynamics. Int. J. Hydrogen Energy.

[4] Long X., Liu Q., Ji B., Lu Y. (2017). Numerical investigation of two typical cavitation shedding dynamics flow in liquid hydrogen with thermodynamic effects. Int. J. Heat Mass Transf..

[5] Zhu J., Zhao D., Xu L., Zhang X. (2016). Interactions of vortices, thermal effects and cavitation in liquid hydrogen cavitating flows. Int. J. Hydrogen Energy.

[6] Naudé C.F., Ellis A.T. (1961). On the mechanism of cavitation damage by nonhemispherical cavities collapsing in contact with a solid boundary. J. Basic Eng..

[7] Arrigoni M., Fuster D., Saini M., Tanne E., Zaleski S. (2022). On the dynamics of a collapsing bubble in contact with a rigid wall. J. Fluid Mech..

[8] Lauer E., Hu X.Y., Hickel S., Adams N.A. (2012). Numerical modelling and investigation of symmetric and asymmetric cavitation bubble dynamics. Comput. Fluids.

[9] Peng C., Tian S., Li G., Sukop M.C. (2018). Single-component multiphase lattice Boltzmann simulation of free bubble and crevice heterogeneous cavitation nucleation. Phys. Rev. E.

[10] Hupfeld T., Laurens G., Merabia S., Barcikowski S., Gökce B., Amans D. (2020). Dynamics of laser-induced cavitation bubbles at a solid–liquid interface in high viscosity and high capillary number regimes. J. Appl. Phys..

[11] Han L., Zhou L., Mukhtar M., Zhang A.M., Han R., Li S. (2025). Influence of hydrostatic pressure on underwater explosion bubble-plate interaction. Appl. Ocean Res..

[12] Yan S., Chen Y., Lyu K., Qin H., Zhang A.M., Li S. (2025). Numerical and experimental benchmark study of nonspherical cavitation bubble dynamics near a rigid wall. Appl. Ocean Res..

[13] Zhang T., Zhang A.M., Zhang S., Long S., Han R., Liu L. (2025). Free-surface jetting driven by a cavitating vortex ring. J. Fluid Mech..

[14] Zhang A.M., Li S.-M., Xu R.-Z., Pei S.-C., Li S., Liu Y.-L. (2024). A theoretical model for compressible bubble dynamics considering phase transition and migration. J. Fluid Mech..

[15] Blake J.R., Gibson D.C. (1987). Cavitation Bubbles Near Boundaries. Annu. Rev. Fluid Mech..

[16] Peng Y., He X., Peng H., Lin Y., Zhang J. (2022). Mesoscopic modeling of vapor cavitation bubbles collapse and interaction in near-wall region with a pseudopotential lattice Boltzmann method. Phys. Fluids.

[17] Wang Y., Peng H., He X., Zhang J. (2022). Cavitation bubbles with a tunable-surface-tension thermal lattice Boltzmann model. Phys. Fluids.

[18] Han R., Li S., Zhang A.M., Wang Q.X. (2016). Modelling for three dimensional coalescence of two bubbles. Phys. Fluids.

[19] Han R., Li S., Tan S., Zhang A.M. (2022). Interaction of cavitation bubbles with the interface of two immiscible fluids on multiple time scales. J. Fluid Mech..

[20] Han R., Zhang A., Liu Y. (2015). Numerical investigation on the dynamics of two bubbles. Ocean Eng..

[21] He X., Peng H., Zhang J., Yuan H. (2022). Multiple vapor cavitation bubble interactions with a thermal lattice Boltzmann method. Ocean Eng..

[22] Rungsiyaphornrat S., Klaseboer E., Khoo B.C., Yeo K.S. (2003). The merging of two gaseous bubbles with an application to underwater explosions. Comput. Fluids.

[23] Chew L.W., Klaseboer E., Ohl S.-W., Khoo B.C. (2011). Interaction of two differently sized oscillating bubbles in a free field. Phys. Rev. E.

[24] Li S., Zhang A.M., Han R., Cui P. (2019). Experimental and numerical study of two underwater explosion bubbles: coalescence, fragmentation and shock wave emission. Ocean Eng..

[25] He X., Peng H., Zhang J., Yuan H. (2023). Thermodynamics of the inception and interactions of multiple laser-produced cavitation bubbles using the lattice Boltzmann method. Comput. Fluids.

[26] He X., Song X., Zhang J., Peng H., Zhou S. (2023). Deciphering surface tension effects of double cavitation bubbles interaction: a lattice Boltzmann study. Int. J. Therm. Sci..

[27] Koukouvinis P., Gavaises M., Georgoulas A., Marengo M. (2016). Compressible simulations of bubble dynamics with central-upwind schemes. Int. J. Computat. Fluid Dynam..

[28] Hsiao C.-T., Ma J., Chahine G.L. (2017). Multiscale tow-phase flow modeling of sheet and cloud cavitation. Int. J. Multiph. Flow.

[29] Ghahramani E., Arabnejad M.H., Bensow R.E. (2019). A comparative study between numerical methods in simulation of cavitating bubbles. Int. J. Multiph. Flow.

[30] Li Q., Luo K.H., Kang Q.J., He Y.L., Chen Q., Liu Q. (2016). Lattice Boltzmann methods for multiphase flow and phase-change heat transfer. Prog. Energy Combust. Sci..

[31] Chen L., Kang Q., Mu Y., He Y.-L., Tao W.-Q. (2014). A critical review of the pseudopotential multiphase lattice Boltzmann model: Methods and applications. Int. J. Heat Mass Transf..

[32] He X., Zhang J., Yang Q., Peng H., Xu W. (2020). Dissolution process of a single bubble under pressure with a large-density-ratio multicomponent multiphase lattice Boltzmann model. Phys. Rev. E.

[33] Peng C., Tian S., Li G., Sukop M.C. (2020). Simulation of laser-produced single cavitation bubbles with hybrid thermal Lattice Boltzmann method. Int. J. Heat Mass Transf..

[34] Chen X.-P., Zhong C.-W., Yuan X.-L. (2011). Lattice Boltzmann simulation of cavitating bubble growth with large density ratio. Comput. Math. Appl..

[35] Sukop M.C., Or D. (2005). Lattice Boltzmann method for homogeneous and heterogeneous cavitation. Phys. Rev. E.

[36] Ezzatneshan E., Vaseghnia H. (2020). Simulation of collapsing cavitation bubbles in various liquids by lattice Boltzmann model coupled with the Redlich-Kwong-Soave equation of state. Phys. Rev. E.

[37] Ezzatneshan E., Vaseghnia H. (2021). Dynamics of an acoustically driven cavitation bubble cluster in the vicinity of a solid surface. Phys. Fluids.

[38] Falcucci G., Jannelli E., Succi S., Ubertini S. (2013). Direct numerical evidence of stress-induced cavitation. J. Fluid Mech..

[39] Peng H., Zhang J., He X., Wang Y. (2021). Thermal pseudo-potential lattice Boltzmann method for simulating cavitation bubbles collapse near a rigid boundary. Comput. Fluids.

[40] Fei L., Qin F., Zhao J., Derome D., Carmeliet J. (2022). Pore-scale study on convective drying of porous media. Langmuir.

[41] Carmeliet J., Derome D., Fei L., Qin F., Zhao J. (2023). Lattice Boltzmann modelling of isothermal two-component evaporation in porous media. J. Fluid Mech..

[42] Li Q., Zhou P., Yan H.J. (2017). Improved thermal lattice Boltzmann model for simulation of liquid-vapor phase change. Phys. Rev. E.

[43] Zhu Y., Shan M., Yang Y., Han Q., Zhu C., Zhang X. (2018). Effect of wettability on collapsing cavitation bubble near solid surface studied by multi-relaxation-time lattice boltzmann model. Appl. Sci..

[44] Shan M., Yang Y., Zhao X., Han Q., Yao C. (2021). Investigation of cavitation bubble collapse in hydrophobic concave using the pseudopotential multi-relaxation-time lattice Boltzmann method*. Chin. Phys. B.

[45] He X., Peng H. (2024). Contact-point analysis of attached-wall cavitation evolution on chemically patterned surfaces using the lattice Boltzmann method. Chem. Eng. Sci..

[46] Wang S.-C., Tong Z.-X., He Y.-L., Liu X. (2022). Unit conversion in pseudopotential lattice Boltzmann method for liquid–vapor phase change simulations. Phys. Fluids.

[47] He X., Peng H. (2024). Modeling inception and evolution of near-wall vapor thermo-cavitation bubbles via a lattice Boltzmann method. Int. J. Hydrogen Energy.

[48] Li Q., Luo K.H., Li X.J. (2013). Lattice Boltzmann modeling of multiphase flows at large density ratio with an improved pseudopotential model. Phys. Rev. E.

[49] Fei L., Du J., Luo K.H., Succi S., Lauricella M., Montessori A. (2019). Modeling realistic multiphase flows using a non-orthogonal multiple-relaxation-time lattice Boltzmann method. Phys. Fluids.

[50] Li Q., Zhou P., Yan H.J. (2016). Pinning–depinning mechanism of the contact line during evaporation on chemically patterned surfaces: a Lattice Boltzmann study. Langmuir.

[51] Hazi G., Markus A. (2009). On the bubble departure diameter and release frequency based on numerical simulation results. Int. J. Heat Mass Transf..

[52] Li Q., Kang Q.J., Francois M.M., He Y.L., Luo K.H. (2015). Lattice Boltzmann modeling of boiling heat transfer: the boiling curve and the effects of wettability. Int. J. Heat Mass Transf..

[53] Yu Y., Li Q., Zhou C.Q., Zhou P., Yan H.J. (2017). Investigation of droplet evaporation on heterogeneous surfaces using a three-dimensional thermal multiphase lattice Boltzmann model. Appl. Therm. Eng..

[54] Chang X., Huang H., Lu X.-Y. (2017). Thermal lattice Boltzmann study of three-dimensional bubble growth in quiescent liquid. Comput. Fluids.

[55] Bremond N., Arora M., Ohl C.-D., Lohse D. (2006). Controlled multibubble surface cavitation. Phys. Rev. Lett..

[56] Dular M., Petkovšek M. (2018). Cavitation erosion in liquid nitrogen. Wear.

[57] Philipp A., Lauterborn W. (1998). Cavitation erosion by single laser-produced bubbles. J. Fluid Mech..

[58] Zwaan E., Le Gac S., Tsuji K., Ohl C.-D. (2007). Controlled cavitation in microfluidic systems. Phys. Rev. Lett..

[59] Bremond N., Arora M., Dammer S.M., Lohse D. (2006). Interaction of cavitation bubbles on a wall. Phys. Fluids.

[60] Li Q., Luo K.H., Kang Q.J., Chen Q. (2014). Contact angles in the pseudopotential lattice Boltzmann modeling of wetting. Phys. Rev. E.

[61] He X., Peng H., Zhang J. (2023). A lattice Boltzmann investigation of liquid viscosity effects on the evolution of a cavitation bubble attached to chemically patterned walls. Phys. Fluids.

[62] Song X., Fei L., Peng H., He X. (2024). Lattice boltzmann investigation of droplet interactions with non-uniform chemically patterned surfaces. Comput. Fluids.

[63] Wen B., Huang B., Qin Z., Wang C., Zhang C. (2018). Contact angle measurement in lattice Boltzmann method. Comput. Math. Appl..

[64] Peng C., Tian S., Li G., Sukop M.C. (2019). Simulation of multiple cavitation bubbles interaction with single-component multiphase Lattice Boltzmann method. Int. J. Heat Mass Transf..

[65] Blake J.R., Robinson P.B., Shima A., Tomita Y. (1993). Interaction of two cavitation bubbles with a rigid boundary. J. Fluid Mech..

[66] Saini M. (2022). Direct Numerical simulations of nucleation and collapse of bubbles attached to wall. Sorbonne Université.

[67] Brujan E.A., Keen G.S., Vogel A., Blake J.R. (2002). The final stage of the collapse of a cavitation bubble close to a rigid boundary. Phys. Fluids.

[68] Peng H., Fei L., He X., Carmeliet J., Churakov S.V., Prasianakis N.I. (2024). Three-dimensional modelling of cavitation bubble collapse using non-orthogonal multiple-relaxation-time lattice Boltzmann method. Ocean Eng..

